# Intense impact of IL-1β expressing inflammatory macrophages in acute aortic dissection

**DOI:** 10.1038/s41598-024-65931-3

**Published:** 2024-06-28

**Authors:** Taishi Inoue, Takuo Emoto, Katsuhiro Yamanaka, Shunya Chomei, Shunsuke Miyahara, Hiroaki Takahashi, Ryohei Shinohara, Takeshi Kondo, Masayuki Taniguchi, Tomoyuki Furuyashiki, Tomoya Yamashita, Ken-ichi Hirata, Kenji Okada

**Affiliations:** 1 Division of Cardiovascular Surgery, Department of Surgery, Kobe University Graduate School of Medicine, 7-5-2 Kusunoki-cho, Chuo-ku, Kobe, 6500017 Japan; 2 Division of Cardiovascular Medicine, Department of Internal Medicine, Kobe University Graduate School of Medicine, Kobe, Japan; 3 Division of Legal Medicine, Department of Community Medicine and Social Healthcare Science, Kobe University Graduate School of Medicine, Kobe, Japan; 4 Division of Pharmacology, Kobe University Graduate School of Medicine, Kobe, Japan; 5 Division of Advanced Medical Science, Kobe University Graduate School of Science, Technology and Innovation, Kobe, Japan

**Keywords:** Aortic dissection, IL-1β, Single cell RNA-sequencing, Cardiology, Cardiovascular diseases

## Abstract

There is no treatment for acute aortic dissection (AAD) targeting inflammatory cells. We aimed to identify the new therapeutic targets associated with inflammatory cells. We characterized the specific distribution of myeloid cells of both human type A AAD samples and a murine AAD model generated using angiotensin II (ANGII) and β-aminopropionitrile (BAPN) by single-cell RNA sequencing (scRNA-seq). We also examined the effect of an anti-interleukin-1β (IL-1β) antibody in the murine AAD model. IL1B^+^ inflammatory macrophages and classical monocytes were increased in human AAD samples. Trajectory analysis demonstrated that IL1B^+^ inflammatory macrophages differentiated from S100A8/9/12^+^ classical monocytes uniquely observed in the aorta of AAD. We found increased infiltration of neutrophils and monocytes with the expression of inflammatory cytokines in the aorta and accumulation of inflammatory macrophages before the onset of macroscopic AAD in the murine AAD model. In blocking experiments using an anti-IL-1β antibody, it improved survival of murine AAD model by preventing elastin degradation. We observed the accumulation of inflammatory macrophages expressing IL-1β in both human AAD samples and in a murine AAD model. Anti-IL-1β antibody could improve the mortality rate in mice, suggesting that it may be a treatment option for AAD.

## Introduction

Acute aortic dissection (AAD) is a life-threatening disease with high morbidity and mortality rates. Algorithms have been designed to constitute treatment options based on the location of the aortic dissection (Stanford type A or B) and acuity. Because type A AAD has an extremely poor natural prognosis with a mortality rate of 0.5–1.0% per hour at 48 h after onset, due to aortic rupture and malperfusion syndromes^1,2^, surgical repair is the only treatment for type A AAD. However, type B AAD have a hospital mortality rate of approximately 10% with antihypertensive agents, which compares favorably with type A AAD^3,4^. Although surgical management is not typically indicated for type B AAD, type B AAD sometimes requires urgent surgical treatment due to rapid expansion, aortic rupture, and malperfusion syndrome^5^. For both types of AAD, no specific medical therapy exists to prevent AAD development or its complications.

Progressive loss of smooth muscle cells in the aortic walls is a crucial feature of AAD and contributes to the weakening of the aortic wall with consequent degeneration. The underlying pathological mechanisms responsible for triggering AAD remain elusive, with the exception of genetic mutations in *FBN1, TGFBR1,* and *TGFBR2*, which cause Marfan and Loeys–Dietz syndromes^6,7^. The depletion and dysfunction of vascular smooth muscle cells (VSMCs) result in the destruction of the extracellular matrix (ECM), leading to the development of AAD^8–10^. Recent studies have revealed that inflammation in the aortic media critically affects VSMCs or the ECM and is key to the pathogenesis of AAD^11^. For example, neutrophils have been reported to contribute to the development and rupture of AAD by promoting MMP-9 expression^12,13^. T cells are also involved in the pathogenesis of AAD; IL-17A plays a pivotal role in aortic wall homeostasis, possibly by modulating TGF-β signaling and ECM metabolism^14^. Regulatory T cells (Tregs) are protective against the incidence of angiotensin II (ANGII)-induced abdominal aortic aneurysm (AAA), although the role of Tregs has not yet been clarified in murine AAD models^15,16^. Macrophages and monocytes play key roles in the development of AAD and AAA^17,18^. Depletion of all aortic macrophages reduces the incidence of aortic rupture, while conditional depletion of Lyve-1^+^ resident macrophages results in increased aortic dilatation in the suprarenal aorta in a murine model using ANGII and β-aminopropionitrile (BAPN)^19^. Another study showed that macrophage-derived Socs3 maintains proper inflammatory responses and differentiation of SMCs, thus promoting fibrotic healing to prevent tissue destruction and AAD development^20^. Because these data suggest that many types of monocytes and macrophages exist in the aorta, the sub-clustering or heterogeneity of monocyte and macrophage populations is important for discovering therapeutic targets.

Single-cell RNA sequencing (scRNA-seq) analysis allows clear identification of monocyte and macrophage heterogeneity, beyond the classical macrophage classification of M1 and M2 phenotypes^21^. A previous study demonstrated that monocytes and macrophages are the major populations in the normal aorta and that the proportions of T cells, B cells, and natural killer (NK) cells are increased in human AAD tissues, although a causal relationship or the therapeutic potential of targeting monocytes and macrophages has not yet been clarified^22^.

In this study, we used scRNA-seq to characterize the specific distribution of myeloid cells in human AAD samples obtained from our facility and in mouse AAD samples from a murine model created by treatment with ANGII and BAPN. Thereafter, we identified therapeutic targets by focusing on the common characteristics of the innate immune system in AAD between humans and the mouse model, and examined the effect of targeting interleukin 1β (IL-1β) as treatment in the mouse AAD model.

## Methods

### Enrollment of study participants

We prospectively enrolled patients with AAD who underwent an aortic replacement in 2022. Two patients with Type A AAD were enrolled for scRNA-seq, and one patient was enrolled for histological analysis. These patients were not diagnosed with Marfan syndrome or Loeys–Dietz syndrome.

**Figure 1:**
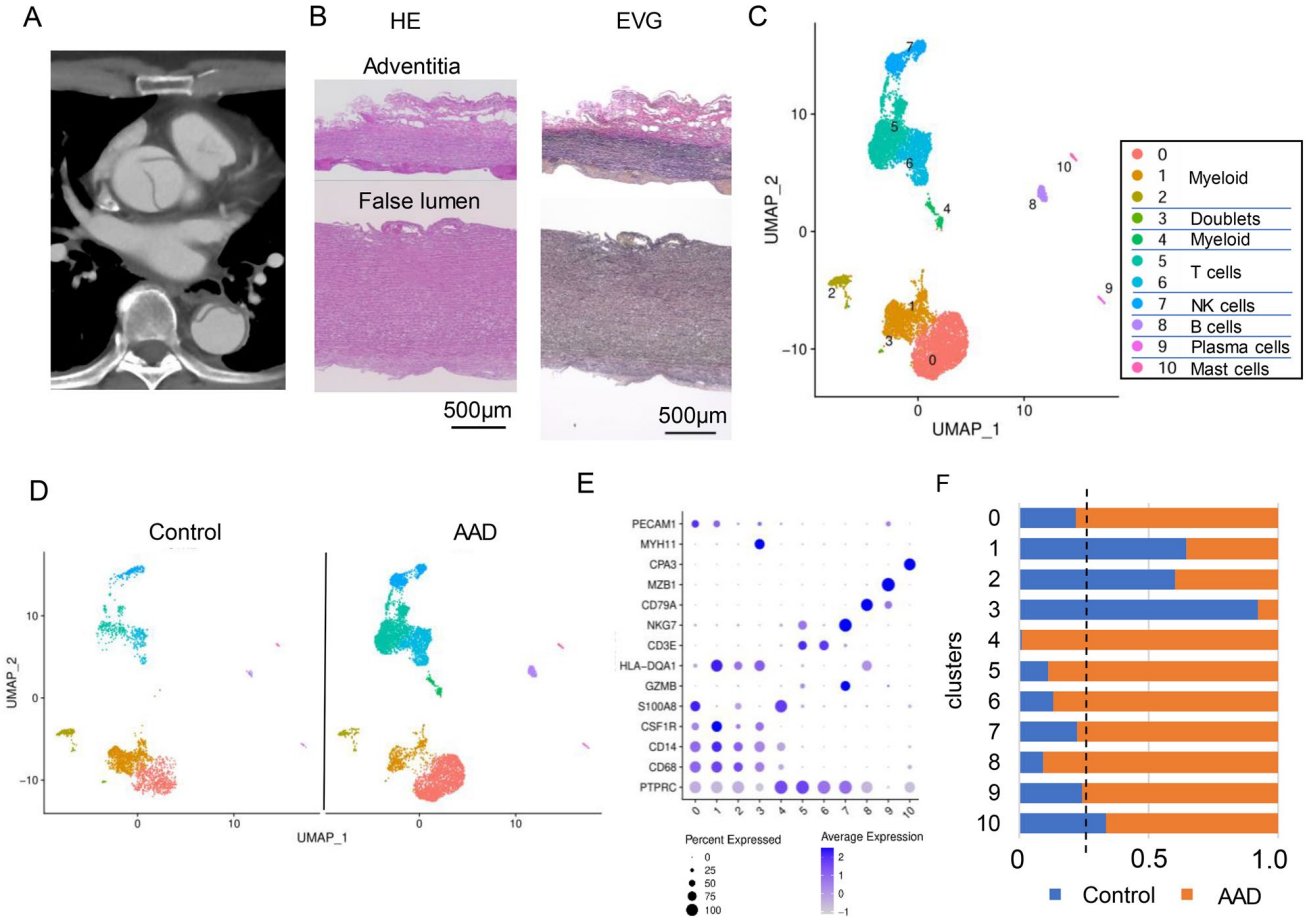
Single-cell RNA sequencing reveals a characteristic immune cell landscape in the ascending aorta in patients with Stanford type A acute aortic dissection (AAD). (**A**) Representative contrast-enhanced computed tomography (CT) image of Stanford type A AAD. (**B**) Histological images stained with hematoxylin and eosin (HE) and Elastica van Gieson (EVG). (**C**,**D**) Uniform manifold approximation and projection (UMAP) dimensionality reduction analysis identifying a unique single-cell immune landscape in the aortas of combined (**C**) and each individual Control or AAD group (**D**) (n = 3 Controls from Li et al.^24^; n = 2 AAD). (**E**) Dot plots displaying signature cell gene expression markers for each immune cell cluster. (**F**) Comparison of the proportions of each cluster between controls and patients with AADs.

All participants provided written informed consent upon enrollment. This study was conducted in accordance with the guidelines of the Declaration of Helsinki and was approved by the Ethics Committee of Kobe University (approval no. B210014). Preoperative computed tomography images are shown in Fig. 1A. Patient characteristics are shown in Table 1.

**Table 1:** Baseline characteristics of study participants.

Variables	AAD1	AAD2	CTRL1 (No.4^a^)	CTRL2 (No.6^a^)	CTRL3 (No.9^a^)
Age, years	70	52	63	61	62
Sex	M	M	F	M	F
Connective tissue disease	No	No	NA	NA	NA
Bicuspid aortic valve	No	No	NA	NA	No
Smoking history	Yes	Yes	No	Yes	Yes
Hypertension	No	Yes	No	Yes	Yes
Diabetes mellitus	No	No	No	No	Yes
Aortic diameter, mm	44	48	NA	NA	22
Rupture	No	No	NA	NA	NA

^a^Control sample data were extracted from a previous study by Li et al.^23^.

### Animal models and interventions

Male C57BL/6J mice were treated with ANGII (angiotensin II acetate salt, 4,006,473.0100, BACHEM, Bubendorf, Switzerland) and BAPN (A3134, Sigma-Aldrich) to induce aortic dissection. ANGII was delivered using subcutaneously implanted mini-osmotic pumps (2002, Alzet, Cupertino, CA, USA) at a dose of 1000 ng/kg/min. BAPN was delivered via drinking water at a concentration of 5 g/l to observe the immune response in the early phase of acute dissection (Figs. 3, 4), and at a dose of 1 g/l to observe the effect of anti- IL-1β antibody (Fig. 5).

#### Anti-IL-1β antibody

A blocking antibody against IL-1β (BE0246, BioXCell Therapeutics, New Haven, CT, USA) or an isotype control antibody (BE0091, BioXCell) were injected intraperitoneally at a dose of 200 µg every 3 days for 2 weeks (Fig. 5).

Experimental procedures followed the institutional guidelines for the care and use of laboratory animals and the ARRIVE guidelines. All experiments were performed according to the Guidelines for Animal Experiments in effect at Kobe University School of Medicine. Animal experiments were approved by the Ethics Committee within Kobe University (approval no. P200706).

### Cell dissociation from human AAD samples and murine AAD samples

#### Human

Resected human AAD samples were immediately stored in a tissue storage solution (130-100-008; Miltenyi Biotec, Bergisch Gladbach, Germany) and processed within 12 h of collection. Each specimen was washed, cut into small pieces, and digested in enzymatic digestion buffer with 3 mg/ml collagenase type II (LS004176, Worthington Biochemical Corp, Lakewood, NJ, USA), 0.15 mg/ml collagenase type XI (H3506, Sigma), 0.25 mg/ml soybean trypsin inhibitor (LS003571, Worthington Biochemical Corp), 0.1875 mg/ml elastase lyophilized (LS002292, Worthington Biochemical Corp), 0.24 mg/ml hyaluronidase type I (H3506, Sigma), and 2.38 mg/ml 4-(2-hydroxyethyl)-1-piperazineethanesulfonic acid (HEPES, H4034, Sigma) in PBS with Ca^2+^ and Mg^2+^, at 37 °C for 60 min while shaking^23^. The mixture was filtered through 40-μm cell strainers (352340, BD Biosciences, Franklin Lakes, NJ, USA), washed, and centrifuged at 300 × *g* for 10 min.

#### Mice

The treated mice were anesthetized with isoflurane at a concentration of 5% and whole aortas from the aortic root to the iliac bifurcation were harvested after whole-body perfusion using 20–30 ml of PBS. Samples were then chopped into small pieces, and digested in enzymatic digestion buffer with 450 U/ml collagenase I (Sigma, C0130), 125 U/ml collagenase XI (Sigma, C7657), 60 U/ml DNase I (Roche, 04536282001), and 60 U/ml hyaluronidase (Sigma, H3506) at 37 °C for 30 min while shaking. The digested samples were filtered through 40-μm cell strainers (BD, 352340), washed, and centrifuged at 3000 × *g* for 10 min.

### Single-cell library preparation and data availability

Single-cell library preparation, data processing, and other methods are described in detail in the Supplemental Methods. All sequencing data are publicly available in the Gene Expression Omnibus (GEO) and can be accessed at GSE.224559. The R code is available from the corresponding author upon request.

## Results

### Single-cell RNA-Seq reveals a characteristic immune landscape of the human aortic wall

We obtained ascending aortic wall tissues from two patients with Stanford type A AAD (Fig. 1A,B), who had not been diagnosed with Marfan syndrome or Loeys-–Dietz syndrome. Normal ascending aortic tissue from two heart transplant recipients and one lung transplant donor was extracted from a previous report^23^. ScRNA-seq was performed using 13,844 cells. These were projected onto a uniform manifold approximation and projection (UMAP). We initially obtained 14 clusters and classified these based on previously reported canonical markers from the genes expressed in each cluster (Supplementary Figs. 1, 2). Inflammatory cell clusters highly expressing *PTPRC* (*CD45*) were extracted from the initially acquired cells, which yielded 9460 cells and 11 clusters (Fig. 1C). We detected five clusters of myeloid cells (clusters 0, 1, 2, 3, and 4, expressing *CD68, CD14, CSF1R,* and *S100A8*), two T cells (clusters 5 and 6, expressing *CD3E*), NK cells (cluster 7, expressing *NKG7*), B cells (cluster 8, expressing *CD79A*), plasma cells (cluster 9, expressing *MZB1*), and mast cells (cluster 10, expressing *CPA3*) (Fig. 1D)^23,24^. The main immune cells within the aortic wall were T cells (48.6%) and myeloid cells (36.1%) (Fig. 1E,F). Interestingly, differences in the composition of clusters were observed between patients with AAD and controls: the proportion of myeloid cells was higher in patients with AAD than in controls.

### Monocytes and inflammatory macrophages expressing IL-1β are accumulated in the ascending aorta in patients with type A AAD

**Figure 2:**
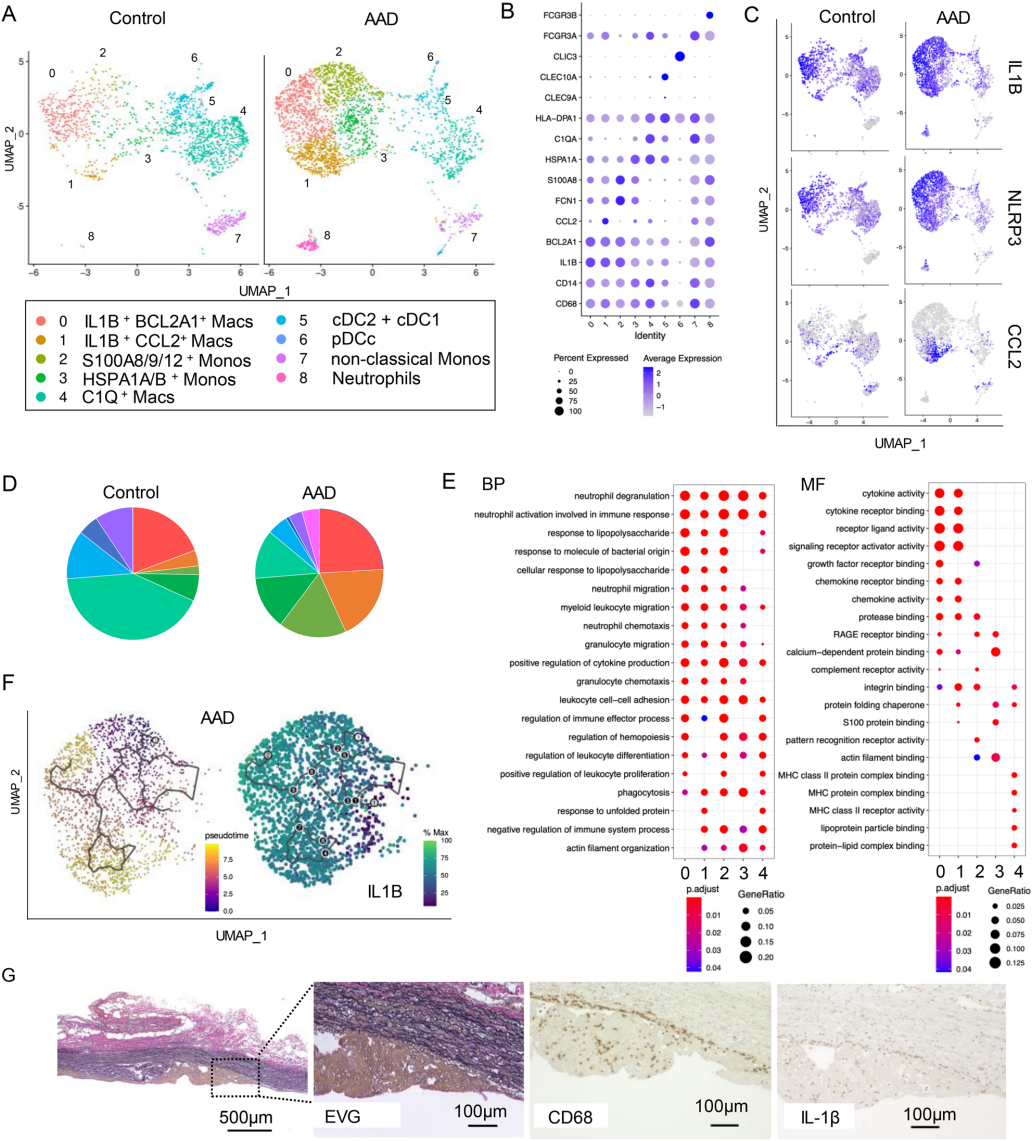
Monocytes and inflammatory macrophages expressing IL-1β are accumulated in ascending aorta in patients with Stanford type A aortic dissection (AAD). (**A**) Sub-clustering of myeloid cells by uniform manifold approximation and projection (UMAP) in Controls and AADs (n = 3 Controls from LiY et al^24^; n = 2 AAD). (**B**) Dot plots displaying signature cell gene expression markers for each subcluster of myeloid cells. (**C**) Featured plots displaying characteristic gene expression of IL1B, NLRP3, and CCL2 in myeloid cells. (**D**) Pie charts showing the proportion of each myeloid cell cluster in the Control and AAD groups. Percentage of partitioned monocytes and monocyte-derived macrophages (clusters 0, 1, 2, and 3). (**E**) Gene ontology (GO) terms showing enriched biological processes (BP) (left) and molecular functions (MF) of clusters 0, 1, 2, 3, and 4. (**F**) Trajectory pseudo-time analysis in Monocle3 with Seurat cluster annotations (left) and change in the expression of IL-1β across pseudo-time for monocytes and monocyte-derived macrophage partitions (clusters 0, 1, 2, and 3) in AAD samples (right). (**G**) Histological staining with EVG, CD68 and, IL-1β. The scale bar represents 100 μm. *Macs* macrophages, *Monos* monocytes, *cDC* conventional dendritic cells, *pDCs* plasmacytoid dendritic cells.

Re-clustering of the myeloid cells revealed nine distinct myeloid clusters, excluding contamination with T-cell doublets: classical monocytes (clusters 2 and 3), non-classical monocytes (cluster 7), macrophages (clusters 0, 1, and 4), dendritic cells (DCs, clusters 5 and 6), and neutrophils (cluster 8) (Fig. 2A). We annotated each cluster from the differentially expressed genes displayed in a heatmap (Supplementary Fig. 3) and the signature cell gene expression markers displayed in feature plots (Fig. 2B,C, Supplementary Fig. 4). Classical monocytes (clusters 2 and 3) expressed *CD14* and *FCN1*. These two clusters were distinguished by *S100A8/9* or heat-shock protein markers, such as *HSPA1A/1B*^21^. The non-classical monocytes expressed higher levels of *FCGR3A* and lower levels of *CD14* than did classical monocytes (cluster 7). Neutrophils (cluster 8) showed low CD14 expression, but high *FCGR3B* (*CD16B*) expression^25^.

“S100A8/9/12^+^ monocytes”, and “HSPA1A/1B monocytes” are reported to be classical monocyte populations detected within the MoMac-VERSE. In this, scRNA-seq datasets from 41 studies are integrated to build a comprehensive view of human myeloid cells, monocyte and macrophage clusters extracted, and reintegrated to generate the MoMac-VERSE^21^. “S100A8/9/12^+^ monocytes” are also detected in coronary atherosclerotic plaques associate with acute coronary syndrome, suggesting that “S100A8/9/12^+^ monocytes” could infiltrate into the aortas^26^. Because neutrophils have a much lower mRNA content and more endogenous RNases than other leukocytes, it is difficult to detect a clear neutrophil cluster in human samples, including in AAD samples^27^.

Macrophages were divided into three clusters: clusters 0 and 1 expressed inflammatory cytokines, such as *IL-1Β, NLRP3, CXCL2*, and *CXCL8*, whereas cluster 4 expressed *C1QA/B/C*, *LYVE1, APOE,* and *TREM2* (Fig. 2B,C, Supplementary Figs. 3 and 4), suggesting that cluster 4 was mixed with resident-type and foamy macrophages^26,28^. Cluster 0 and 1 were both inflammatory and were distinguished by the expression levels of *BCL2A1* and *CCL2*. Therefore, we defined cluster 0 as IL-1Β^+^ BCL2A1^+^ inflammatory macrophages and cluster 1 as IL-1Β^+^ CCL2^+^ inflammatory macrophages. BCL2A1 (B-cell lymphoma 2-related protein A1) is important in the hematopoietic system and exerts its anti-apoptotic function by sequestering pro-apoptotic BCL2 proteins^29^. Nuclear factor kB (NF-kB) is an important inducer of BCL2A1 expression. CCL2 is a chemokine to induce accumulation of classical monocytes expressing CCR2 and reported to be involved in the formation of atherosclerosis^30^. IL-1Β^+^ CCL2^+^ inflammatory macrophages (cluster 1) were characteristically observed in the aortas of AAD, which suggested a positive loop of the recruitment of classical monocytes expressing CCR2. Next, we identified two clusters of DCs that showed high expression of human leukocyte antigens (HLAs) such as *HLA-DR*, *HLA-DQ*, and *HLA-DP*: conventional DC1 (cDC1) expressing CLEC9A and CADM1 (cluster 5); cDC2 expressing CLEC10A and CD1C (cluster 5); and plasmacytoid DC expressing GZMB and CLIC3 (cluster 6) (Fig. 2B, Supplementary Figs. 3 and 4).

The proportion of myeloid cell clusters was dynamically altered: C1Q^+^ macrophages (cluster 4), supposed to be resident-type and foamy macrophages, occupied the largest population in the controls, whereas the proportions of classical monocytes and monocyte derived macrophages including IL-1Β^+^ BCL2A1^+^ (cluster 0), IL-1Β^+^ CCL2^+^ inflammatory macrophages(cluster 1), S100A8/9/12^+^ classical monocytes (cluster 2), and HSPA1A/B^+^ monocytes (cluster 3) were increased in patients with AAD (Fig. 2D). Gene Ontology (GO) enrichment analysis showed that cytokine activity, cytokine receptor binding, and chemokine and chemokine receptor binding were enriched in IL-1Β^+^ BCL2A1^+^ (cluster 0) and IL-1Β^+^ MARCO^+^ inflammatory macrophages (cluster 1) in terms of molecular function terms and that responses to lipopolysaccharide and molecules of bacterial origin were enriched in terms of biological process terms. In contrast, C1Q^+^ macrophages (cluster 4) showed high antigen-presenting activity via MHC class II and lipoprotein metabolism (Fig. 2E). Pseudo-time trajectory analysis demonstrated that S100A8/9/12^+^ classical monocytes were differentiated in three directions: IL-1Β^+^ BCL2A1^+^ and IL-1Β^+^ CCL2^+^ inflammatory macrophages with higher levels of *IL1Β* expression, and HSPA1A/B^+^ monocytes with lower levels of *IL1Β* expression (Fig. 2F). Histological analyses of the AAD samples revealed that inflammatory macrophages stained for both IL-1β and CD68 had accumulated on the side of the false lumen of the adventitia (Fig. 2G).

### Central memory CD4^+^ T cells are found in patients with type A AAD

T cells were the second major cell population in these samples, accounting for 36.1% of all immune cells. The proportion of T cells was higher in AAD samples (Fig. 1F). Re-clustering of T cells revealed three CD4^+^ and four CD8^+^ T cell populations (Supplementary Fig. 5A). Thereafter, re-clustering of CD4^+^ T cells revealed four distinct sub-clusters: effector CD4^+^ T cells expressing higher levels of *CD69* and *CCL5* (cluster 0)^24^; naïve CD4^+^ T cells expressing the same level of *IL7R* and lower levels of *CD69* than cluster 0 (cluster 1); central memory CD4^+^ T cells expressing *CCR7*, *LEF1*, *SELL, LTB,* and *KLF2* (cluster 2)^31–33^; and regulatory CD4^+^ T cells expressing *FOXP3* and *IL2RA* (cluster 3) (Supplementary Fig. 5B–D)^31^. The proportions of naïve and central memory CD4^+^ T cells were higher in patients with AAD (Supplementary Fig. 5B–D). Re-clustering of CD8^+^ T cells revealed four sub-clusters: effector CD8^+^ T cells expressing *GZMK* and *CD69*^34^, GZMB^+^ cytotoxic CD8^+^ T cells expressing *GZMB* and *PRF1,* and IFNg^+^ effector memory CD8^+^ T cells. The proportion of GZMB^+^ cytotoxic CD8^+^ T cells was higher in AAD samples (Supplementary Fig. 5E–G).

### Infiltration of neutrophils and monocytes into the aorta is observed before macroscopic dissection in a murine AAD model generated using ANGII and BAPN

**Figure 3:**
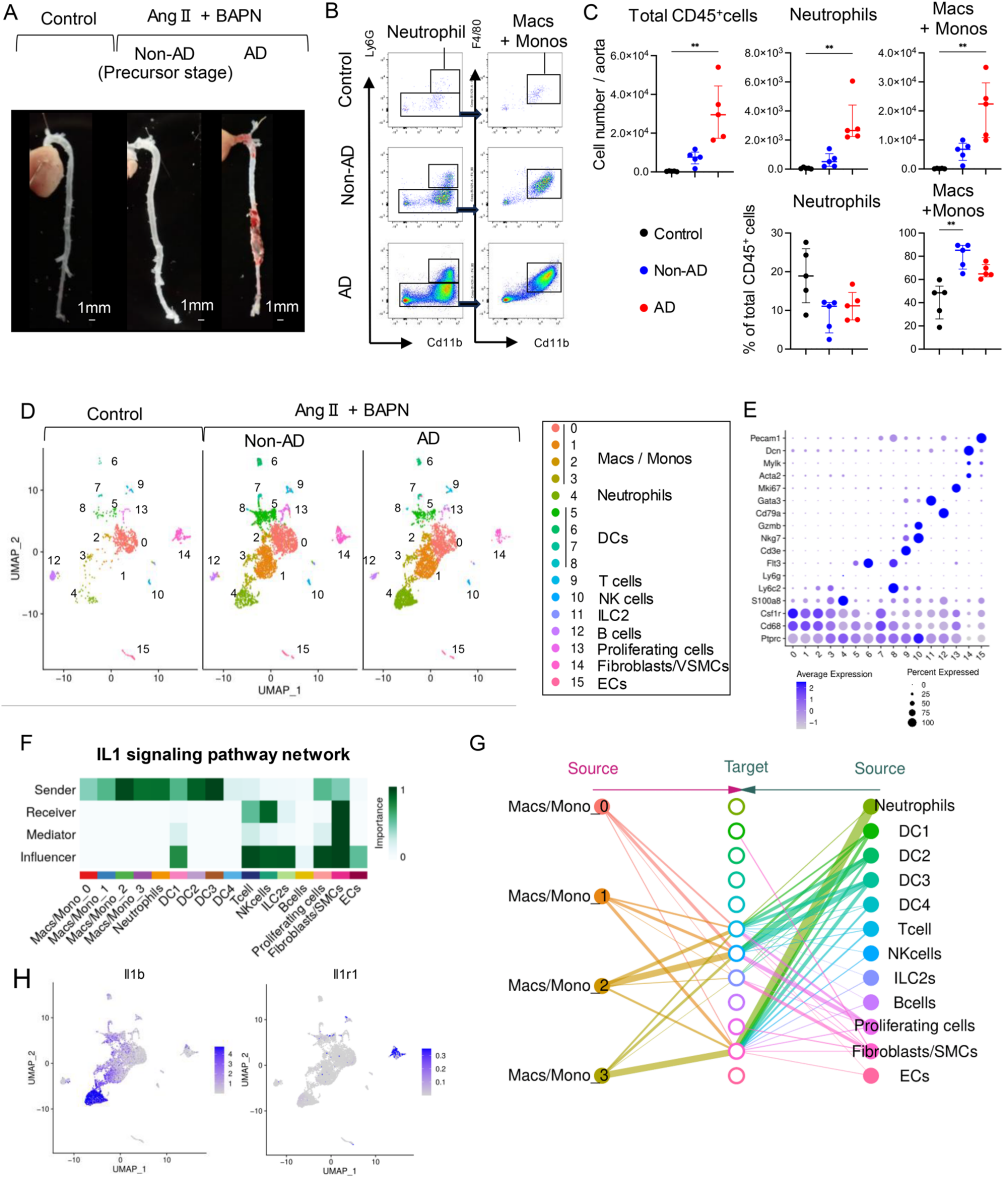
In mice, treatment with angiotensin II (Ang II) and β-aminopropionitrile (BAPN) causes acute inflammation before aortic dissection occurs. (**A**) Gross morphology of aortic dissection (AD) in male C57BL/6J mice; aortas in control mice without Ang II or BAPN treatment (Control), aortas without AD after exposure to ANGII (1 µg/kg/µl) and BAPN (5 g/drinking water) (No-AD), and aortas with AD after exposure to ANGII and BAPN (AD). (**B**,**C**) Flow cytometric analysis showing percentages with plots after gating of CD45^+^ immune cells excluding doublets (**B**) and cell numbers of total CD45^+^ cells, neutrophils, Ly6C^high^ monocytes, and macrophages (**C**). (n = 5 controls, n = 4 Non-AD, n = 5 AD). One-way analysis of variance with Tukey’s multiple comparison test; *P < 0.05. (**D**) Uniform manifold approximation and projection (UMAP) dimensionality reduction analysis identifying unique immune populations in male mice exposed to Ang II (1 µg/kg/µl) and BAPN (5 g/l in drinking water) for 1 week. (**E**) Dot plots displaying the signature cell gene expression markers. (**F**,**G**) CellChat showing the activated interaction between each cell population in the interleukin-1 (IL-1) signaling pathway. (**H**) Feature plots of IL-1β and Il1r1 expression on UMAP. CellChat; a tool that can quantitatively infer and analyze intercellular communication networks from scRNA-seq data. *Macs* macrophages, *Monos* monocytes, *DCs* dendritic cells, *NK cells* natural killer cells, *ILCs* Innate lymphoid cells, *VSMCs* vascular smooth muscle cells, *ECs* endothelial cells.

To discover immune targets for treating AAD, we used a well-established murine AAD model treated with ANGII (1000 ng/kg/min) and BAPN, an inhibitor of lysyl oxidase (5 g/L in drinking water). To verify whether the inflammatory cells observed in human tissues were responsible for the development of AAD, we used scRNA-seq to compare the immune cell landscape of murine ascending to thoracic aorta among the three groups: a control group without any intervention (Control), a group without AAD after administration of ANGII and BAPN, as a non-AD group, and a group with AAD development after administration of ANGII and BAPN, as an AD group (Fig. 3A). Due to early sacrifice after drag intervention, the non-AD group was presumed to represent a precursor stage to the AD group. Flow cytometry of day 7 aortic tissues revealed a significant increase in the number of CD45^+^ immune cells, neutrophils and macrophages, respectively in the AD group. The proportion of macrophages significantly increased in the non-AD group, whereas that of neutrophils did not increase (Fig. 3B,C). Using scRNA-seq for a more detailed examination of the differences in cell populations, we obtained 8816 cells and 16 clusters (Fig. 3D). The clusters were annotated using previously reported canonical markers (Fig. 3E, Supplementary Fig. 6)^27,35^. The populations of myeloid cells (clusters 0–3) were markedly increased in the non-AD and AD groups as compared to the control group, regardless of the occurrence of AAD.

CellChat is a tool that is able to quantitatively infer and analyze intercellular communication networks from scRNA-seq data^36^. In order to predict signaling inputs and outputs of each single cell using network analysis and pattern recognition approaches, we applied our data to CellChat. Based on CellChat analysis related to IL-1β including all three groups, we found that myeloid cells mainly sent IL1 signaling, while non-inflammatory cells, including VSMCs and fibroblasts, mainly received IL1 signaling (Fig. 3F,G). Indeed, *Il1b* expression was particularly prominent in myeloid cells, whereas *Il1r1*, which encodes the major receptor for IL-1β, was specifically expressed in clusters of VSMC and fibroblasts (Fig. 3H). Therefore, IL1 signaling-mediated inflammatory responses appear within the aortic wall even before the onset of AD.

### Differentiation from classical monocytes into IL-1β^+^ inflammatory macrophages is a therapeutic target to prevent death due to AAD

**Figure 4:**
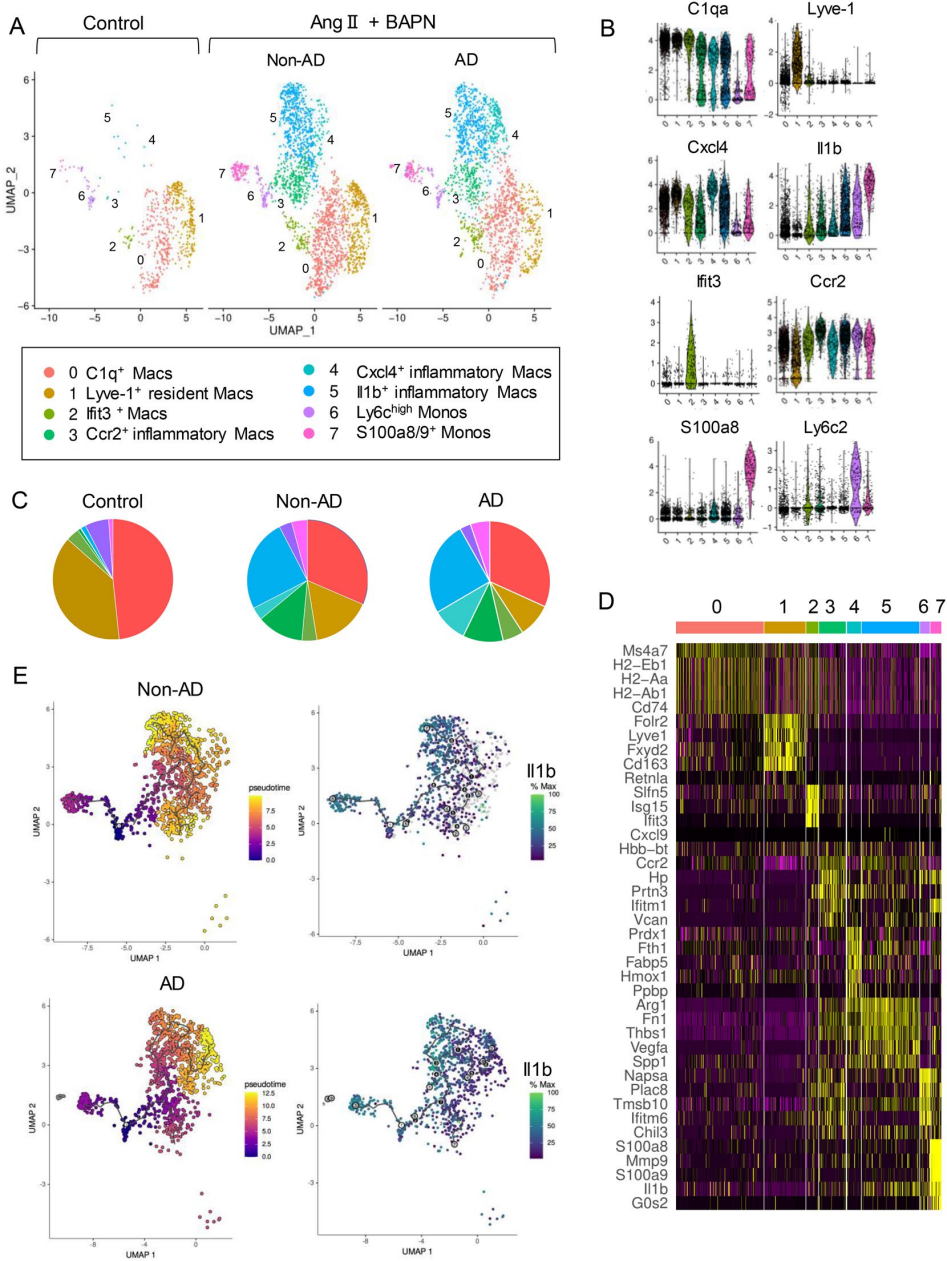
IL-1β^+^ inflammatory macrophages are accumulated before the onset of macroscopic aortic dissection (AD) in mice. (**A**) Re-clustering of monocytes and macrophages into three groups: mouse aortas without Ang II or BAPN (control), mouse aortas without AD after exposure to Ang II and BAPN (non-AD), and mouse aortas with AD after exposure to Ang II and BAPN (AD). (**B**) Violin plots depicting single-cell gene expression of each canonical monocyte or macrophage marker for clustering. (**C**) Proportion of each monocyte-macrophage cluster. (**D**) Heatmap of the top-5 differentially expressed genes in each monocyte and macrophage cluster. (**E**) Trajectory pseudo-time analysis in Monocle3 with Seurat cluster annotations (left) and change in the expression of IL-1β across pseudo-time for the monocyte and monocyte-derived macrophage partitions (clusters 0, 1, 2, 3, 4) in the non-AD and AD groups (right). *Macs* macrophages, *Monos* monocytes, *Ang II* Angiotensin II, *BAPN* β-aminopropionitrile.

We extracted clusters of monocytes and macrophages to verify the presence of resident type and inflammatory macrophages, observed in human samples. We identified two monocyte clusters and six macrophage clusters, and named these sub-clusters based on the gene marker characteristics of each cluster (Fig. 4A,B). Clusters 0 and 1 were the main clusters in the control group (Fig. 4C). Cluster 1 was clearly defined as resident macrophages due to the high expression of *Lyve1* and *Folr2* (Fig. 4D). Cluster 0 is considered to be resident-like macrophages, as it exhibits a low level of *Lyve1* expression, while also possessing markers of diverse M2-like macrophage functions, such as *Cd74*^35^. Cluster 2 prominently expressed *Ifit3* and *Isg15,* which are genes involved in type I interferon signaling, implying that these were interferon-inducible macrophages^27^. Therefore, we named cluster 0 as C1q^+^ macrophages, cluster 1 as Lyve-1^+^ resident macrophages, and cluster 2 as Ifit3^+^ macrophages.

Clusters 3–5 were inflammatory macrophages specific to AD models; each cluster uniquely expressed inflammatory cytokine genes such as *Ccr2* (cluster 3), *Cxcl4* (cluster 4), and *Il1b* (cluster 5). Interestingly, cluster 5 also showed a high expression of *Arg1*, a marker of M2 macrophages (Fig. 4D). Therefore, unlike previous studies, we found no clusters that strictly resembled typical M1/M2 polarized macrophages^37^. In the trajectory analysis, inflammatory macrophages (clusters 3, 4, and 5) were recognized as a series of macrophages existing within the same differentiation lineage from Ly6c^high^ monocytes (cluster 6). Furthermore, Ly6c^high^ monocytes (cluster 6) differentiated into Ccr2^+^ macrophages (clusters 3), and then differentiated into Il1b^+^ macrophages (cluster 5). When comparing AD group to non-AD group, both groups had differentiation from Ly6c^high^ monocytes to Il1b^+^ macrophages. However, uniquely in the AD group, there was an additional differentiation observed from Il1b^+^ macrophages to Cxcl4^+^ macrophages (Fig. 4E). We observed the consistent response of innate immune system between human and mice; resident macrophages occupied the majority of macrophage population in the murine control group and in human control samples, while Il1b^+^ inflammatory macrophages uniquely appeared in the murine non-AD precursor group and AD groups and in human AAD patients.

### IL-1Β neutralizing antibody increases aortic elastin contents

**Figure 5:**
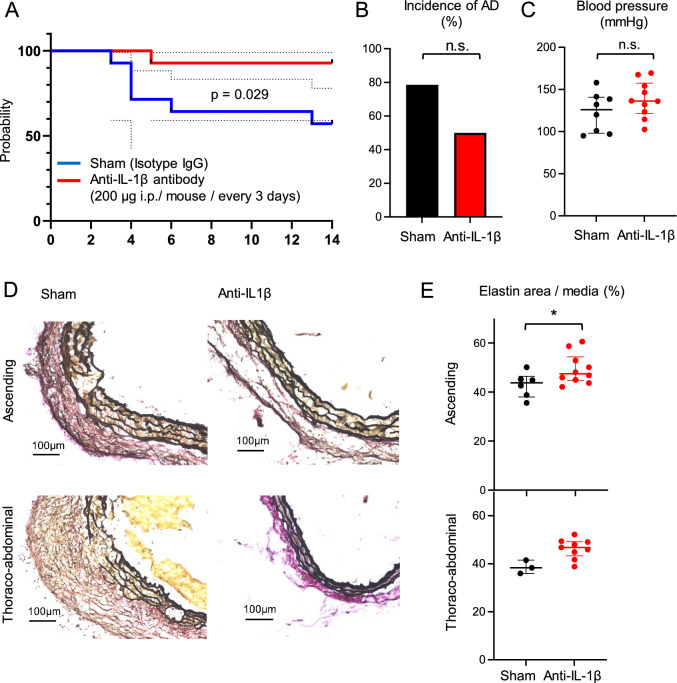
Anti-IL-1β neutralizing antibody improves survival rate in mice. (**A**) Kaplan–Meier survival curve tracking death due to ruptured aortic dissection (AD) in male C57BL/6J mice exposed to Ang II (1 µg/kg/µl) and BAPN (1 g/l in drinking water) for 2 weeks after treatment with isotype control or anti-IL-1β neutralizing antibody (isotype IgG or anti-IL-1β neutralizing antibody; 200 β g i.p./mouse/every 3 days, n = 14 sham; n = 14 anti-IL-1β neutralizing antibody). Log-rank (Mantel–Cox) test, *P < 0.05. (**B**) Incidence of AD in AD model mice treated with the isotype control or anti-IL-1β antibody (n = 11/14 sham; n = 7/14 anti-IL-1β neutralizing antibody). (**C**) Blood pressure of AD model mice treated with the isotype control or anti-IL-1β neutralizing antibody (n = 8, sham; n = 9, anti-IL-1β neutralizing antibody). (**D**) Representative images of Elastica van Gieson (EVG) staining in the ascending (upper) and thoracoabdominal aorta (bottom). (**E**) Percentage of EVG-stained area per total tunica media without AD in the ascending aorta (upper) and thoracoabdominal aorta (bottom). *P < 0.05. *Ang II* Angiotensin II, *BAPN* β-aminopropionitrile.

To investigate the association between IL-1β mainly derived from inflammatory macrophages, we conducted blocking experiments using anti-IL-1β antibody. The survival rate associated with aortic rupture was 60% in the sham group vs. 90% in the IL-1β neutralization group at 14 days (p = 0.029) (Fig. 5A). The incidences of aortic rupture and death were significantly reduced by anti-IL-1β neutralization, although the incidence of AAD did not differ significantly (Fig. 5B). No significant difference in blood pressure was observed between the two groups (Fig. 5C).

To investigate the underlying effect of anti-IL-1β antibodies, we examined the histological differences in elastin degradation between the ascending aorta and the suprarenal thoracoabdominal aorta, which are common sites of aortic dissection in each group. The areas occupied by elastin within the aortic wall were significantly increased by administration of anti-IL-1β antibody (Fig. 5D,E). This finding suggested that administration of an IL-1β antibody inhibited the inflammatory destruction of elastin structures induced by BAPN and ANGII. The results are summarized in Supplementary Fig. 7.

## Discussion

It has been challenging to understand the molecular pathology of human AAD because of the rapid destruction of the aortic walls and the associated high mortality. For the first time, we showed the detailed characteristics of aortic immune cells with AAD both in human and mice using scRNA-seq and found IL-1β as a therapeutic target for AAD.

In detail, we found that the proportion of classical monocytes and monocytes derived macrophages expressing IL1B were higher in patients with AAD than in controls. Trajectory analysis showed that the recruited monocytes differentiated into macrophages that expressed more *IL1Β* than did the classical monocytes. Although this is consistent with the previous report^22^, we, for the first time, clearly showed the detailed characteristics of monocyte macrophage population by scRNA-seq. To clarify the causal involvement of the innate immune system, we used a well-established AAD model treated with BAPN and ANG2. To obtain information on the aortas of the precursor stage of AAD, we used a high dose of BAPN (5 g/L) to increase the incidence of AAD and evaluated the immune response at an early time point. We found increased infiltration of monocytes into the aortas and accumulation of inflammatory macrophages derived from infiltrating monocytes prior to the onset of macroscopic AAD, whereas further differentiation from Il1b^+^ macrophages into Cxcl4^+^ macrophages occurred after the onset AAD. Resident type of macrophages occupied the majority of macrophage population in the murine control group and in the human control samples, while inflammatory macrophages uniquely appeared in the murine non-AD group and AD groups and in patients with AAD. These dynamic changes were not only due to the condition of the result of AAD, but also contributed to the pathology of AAD including initiation, worsening and rupture. To validate these results and explore minimally invasive treatment options in the clinical setting, we administered an anti-IL-1β antibody to block IL-1 signaling in a mouse model of AAD. Although anti-IL-1β antibody could not significantly suppressed the incidence of AAD, it suppressed death due to aortic rupture, suggesting that anti-IL-1β could be a treatment option for patients with AAD to prevent death from aortic rupture.

ScRNA-seq data explained the mechanism why classical monocytes were accumulated in patients with AAD. IL1B^+^ CCL2^+^ macrophage express high level of CCL2 and recruited CCR2^+^ expressing monocytes and formed a positive feedback loop of monocyte macrophages expansion in the aortic tissue with AAD. Anti-apoptotic function of BCL2A1 explained that IL-1Β^+^ BCL2A1^+^ inflammatory macrophages were detected even in controls, although classical monocytes which should be the source of IL-1Β^+^ BCL2A1^+^ inflammatory macrophages occupied a small proportion.

The proportions of both CD4^+^ and CD8^+^ T cells were increased in human AAD. In particular, central memory CD4^+^ T cells were observed only in AAD samples, suggesting that the acquired immune system may also be involved in the pathogenesis of AAD. While we and other groups had previously found TREM2^+^ foamy macrophages to be present in atherosclerotic plaques^26,31^, we could not detect TREM2^+^ macrophages in human AAD samples, suggesting that human AAD is not related to atherosclerotic plaques.

The tunica media of the aortas is mainly composed of VSMCs, proteoglycans, elastic fibers, and collagen fibers^11^. BAPN was administered to this model to inhibit collagen and elastin cross-linking. CellChat analysis revealed that monocytes, macrophages, and neutrophil-derived IL-1β stimulate VSMCs or fibroblasts expressing IL1 receptors. We should focus on the results showing that neutrophils strongly expressed Il1b (Fig. 3H) and exhibit robust interactions with SMCs (Fig. 3G). However, as previously demonstrated by Anzai et al., the interactions involving neutrophils predominantly occur after the onset of AAD^13^. Prior to the onset, our flow cytometry data showed that neither the absolute number of neutrophils within the aorta nor their proportion among inflammatory cells was increased (Fig. 3C). On the other hand, macrophages demonstrated moderate interactions with SMCs (Fig. 3G) and a significant pre-onset increase in their proportion among inflammatory cells. Furthermore, it was the inflammatory macrophages that were notably increasing (Fig. 4A). Therefore, we consider that Il1b-positive macrophages are the primary contributors to elastin degradation even before the onset of AAD.

Immunohistochemistry showed that CD68^+^ and IL-1β^+^ macrophages accumulated at the site of human AAD. These findings suggested that IL-1 signaling could affect the pathology or severity of AAD. Johnston et al. had already reported that increased IL-1β protein levels were detected in human thoracic aortic aneurysm (TAA) as compared to healthy aortas^38^. Considering that TAA is a precondition of AAD^39,40^, the development of AAD seems to be driven by a similar inflammatory process, with or without an aortic aneurysm. In terms of the mechanism of down-stream of IL1 receptors, Guo et al. showed that IL-1β-mediated inflammation induced MMP2 and MMP9 expression, as well as apoptosis of VSMCs, and led to destruction of elastin structures in a rat AAD model^41^. The present study demonstrated that anti-IL-1β antibody inhibited destruction of elastin structures by BAPN and ANGII, although the exact mechanisms by which IL-1β leads to development of AAD remain unclear and require elucidation in future.

Clinically, anti-IL-1β antibodies, named canakinumab, have been shown to lead to a significantly lower rate of recurrent cardiovascular events without noteworthy complications, as compared to placebo, in patients with a history of myocardial infarction^42^. In an aged society, patients with AAD are now older than before, and many patients cannot undergo surgical aortic repair because of complications. Treatment with an anti-IL-1β antibody may be an alternative to surgical aortic repair for high-risk patients. In addition, it would be possible to treat patients with AAD by anti-IL-1β antibody immediately after diagnosis, in order to relieve the inflammation of the aortic wall, and to provide more time for preparing for surgical aortic repair.

Our study includes some limitations. First, we do not have our own human control dataset obtained in our facility. However, the consistent findings about monocytes and macrophages between human and mice supported the reliability of comparison using control samples from open-source dataset in human. Secondly, we could not detect non-immune cells such as smooth muscle cells or endothelial cells in human AAD samples by scRNA-seq. Thirdly, we could not clearly show the down-stream molecular mechanism of the effect of IL-1β involved in the pathology of AAD.

In conclusion, using scRNA-seq, we here revealed the accumulation of inflammatory macrophages expressing IL-1β in both human patients with AAD and in a murine AAD model. Treatment with anti-IL-1β antibodies could improve the mortality rate of murine AAD, suggesting that this could be an option for treating AAD, particularly in high-risk patients; this warrants further investigation in clinical trials.

### Supplementary Information

Supplementary Information.

Supplementary Figures.

## Abbreviations

AAD: Acute aortic dissection
ANGII: Angiotensin II
BAPN: β-Aminopropionitrile
ECM: Extracellular matrix
IL-1β: Interleukin-1β
scRNA-seq: Single-cell RNA sequencing
Tregs: Regulatory T cells
VSMCs: Vascular smooth muscle cells
