# Supplementary material for: Intense impact of IL-1β expressing inflammatory macrophages in acute aortic dissection
Source: Sci Rep. 2024 Jun 28;14:14893. doi: 10.1038/s41598-024-65931-3 (PMC11211506; doi:10.1038/s41598-024-65931-3)
Supplement: Supplementary file 1 — Supplementary Information. [file 41598_2024_65931_MOESM1_ESM.docx]

**Supplemental Methods**

**Histology**

*Human:* AAD samples were immediately fixed with Paraformaldehyde, 4% in PBS. Tissue specimens were processed with a paraffin embedding system. AAD sections were cut into a thickness of 4 µm, mounted on slides, and stained with Hematoxylin and eosin and Elastic-Van Gieson (EVG) (HT25A, Sigma). For immunohistochemistry, LA sections were incubated with the primary antibodies to CD68 (1:500, Agilent Technologies, M0814), IL1B (Santa Cruz, sc-7884), overnight at 4°C, followed by the HRP conjugated anti-mouse IgG(H+L) goat polyclonal secondary antibody (HISOFINE #424134; Nichirei Bioscience, Tokyo, Japan) for 30 minutes at room temperature. The staining was visualized using 3,3'-diaminobenzidine, tetra-hydrochloride (DAB). Nuclear was identified with hematoxylin.

*Mice:*  Whole aortas from the aortic root to the iliac bifurcation were collected from mice after whole-body perfusion using 20-30ml of PBS. Aortas were then sectioned and either immediately embedded in O.C.T. compound (Sakura, Finetek) and flash-frozen in a fixative 4% paraformaldehyde for a minimum of 3 hours, and up to 24 hours, prior to embedding. Serial cross sections (10 µm) were cut using a cryostat and air dried onto microscope slides (Fisher Scientific, Pittsburgh, PA). The suprarenal aorta (region below the diaphragm and above the renal arteries were sectioned into 10μm slices. All-in-one–type fluorescence microscope (BZ-X700; Keyence) were used for observation of stained sections.

**EVG evaluation**

Percentages of EVG stained area per total tunica media area were measured by Image J. The samples with AAD were excluded from this analysis.

**Flow Cytometry.**

Antibodies used for flow cytometric analyses are provided in Supplementary Table 2. Data was acquired on an LSRII flow cytometer (BD Biosciences) and analyzed with FlowJo v8.8.6 (Tree Star, Inc.). Digested aortic cells were treated with FcBlock (BD Biosciences) for 15 min before incubation with antibody cocktail for an additional 30 min.

**Single-cell sequencing library preparation**

Single cell sequencing libraries were prepared as outlined in the 10x Genomics Single Cell 3′ Reagent Kits v3.1 user guide. Before loading onto the 10x Genomics single-cell-G chip, the cell concentration was adjusted to reach the required cell concentration according to the user guide by pelleting and re-suspending the samples. The cell suspensions were loaded onto a Chromium single-cell chip along with partitioning oil, reverse transcription (RT) reagents, and a collection of gel beads that contained unique 10X barcodes.

After the preparation of single-cell gel bead-in-emulsions (GEMs), reverse transcription (RT) was performed using a Thermal Cycler (Thermo‌ ‌Fisher‌ ‌Scientific). Samples were diluted and run on a Bioanalyzer (Agilent Technologies) to determine the concentration of cDNA. cDNA libraries were prepared as outlined by the Single Cell 3′ Reagent Kits v3.1 user guide by modifying the PCR cycles appropriately based on the calculated cDNA concentration (as recommended by 10X Genomics).

The amplified cDNA was purified by using SPRI select magnetic beads (Beckman Coulter). Single cell libraries were then constructed by fragmentation, end repair, polyA tailing, adaptor ligation, and size selection based on the manufacturer’s standard parameters. Each sequencing library was created with a unique sample index.

**Single cell RNA sequencing data processing**

The sequenced data were processed into expression matrices with the Cell Ranger Single Cell Software (v 6.1.2) of 10X Genomics against the GRCh38 human reference genome or the mm10 murine reference genome with default parameter. Raw base call file from Novaseq sequencer were demultiplexed into FASTQ files for each library. Analyses were performed using Seurat (v.4.1.1) in an R 4.2.1 environment. Before processing, reads were filtered to remove mitochondrial genes. To exclude doublets and low-quality cells, only cells expressing between 500 and 5,000 genes and less than 8% mitochondrial genes and genes expressed in at least 3 cells were used for further analyses. As controls, we utilized openly published datasets of single cells obtained from healthy aortas.^1^ The two datasets (AAD and Control groups) were merged, Log-normalized with the default parameters. After removing non-immune cells such as endothelial cells, fibroblasts, and smooth muscle cells, we clustered all the cells based on the integrated gene expression matrix. For the visualization of the cells in a two- dimensional space, we performed a principal component analysis on the integrated dataset and used the first 30 principal components (PCs) for uniform manifold approximation and projection (UMAP).

To assess differential gene expression, we performed non-parameteric Wilcoxon rank sum test implemented by Seurat *FindMarkers* function. Top10 unique differentially expressed genes in each cluster were identified by testing gene expression between each cluster and all other clusters combined and displayed as heatmaps by using Seurat *Doheatmap* function. Violin plots or Featured plots were performed to identify each cluster based on the unique differentially expressed genes or the expression of the known canonical marker genes. Next, we extracted each cluster from the integrated dataset and re-clustered them by the same procedure.

**GO (Gene ontology) analyses**

We applied the cluster Profiler package to use gene ontology（GO）analyses for biological process based on the selected differentially expressed genes in each macrophage (including classical monocyte) cluster.

**Monocle trajectory analyses**

Monocle3 (v.1.2.9) was used to generate the pseudo-time trajectory analysis. UMAP embeddings and cell subclusters generated from Seurat were converted to a celldata set object using *SeuratWrappers* (v.0.2.0) and then used as input to perform trajectory graph learning and pseudo-time measurement.

**CellChat**

Cell-cell interaction analysis was performed using CellChat (version 1.6.0).^2^ IL1 signaling pathway network was evaluated by CellChat.

**Statistics.**

Results are expressed as mean ± SEM. The unpaired t-test was used to evaluate the differences between two groups The GraphPad Prism 9 software was used for all the statistical analyses. P values of 0.05 or less were considered to denote significance.

1 Li, Y. *et al.* Single-Cell Transcriptome Analysis Reveals Dynamic Cell Populations and Differential Gene Expression Patterns in Control and Aneurysmal Human Aortic Tissue. *Circulation* **142**, 1374-1388, doi:10.1161/CIRCULATIONAHA.120.046528 (2020).

2 Jin, S. *et al.* Inference and analysis of cell-cell communication using CellChat. *Nat Commun* **12**, 1088, doi:10.1038/s41467-021-21246-9 (2021).

**Supplemental Table**

| Target | Fluorophore/ Conjugate | Company | Clone | Catalogue Number |
| --- | --- | --- | --- | --- |
| F4/80 | BV421 | BD biosciences | T45-2342 | 565411 |
| Ly6C | PE-Cy7 | Biolegend | HK1.4 | 128018 |
| Ly6G | PE | BD biosciences | 1A8 | 551461 |
| Cd11b | BV650 | BD biosciences | M1/70 | 563402 |
| Cd45 | APC-Cy7 | BD biosciences | 30-F11 | 557659 |
