# Supplementary material for: Intense impact of IL-1β expressing inflammatory macrophages in acute aortic dissection
Source: Sci Rep. 2024 Jun 28;14:14893. doi: 10.1038/s41598-024-65931-3 (PMC11211506; doi:10.1038/s41598-024-65931-3)
Supplement: Supplementary file 2 — Supplementary Figures. [file 41598_2024_65931_MOESM2_ESM.pdf]

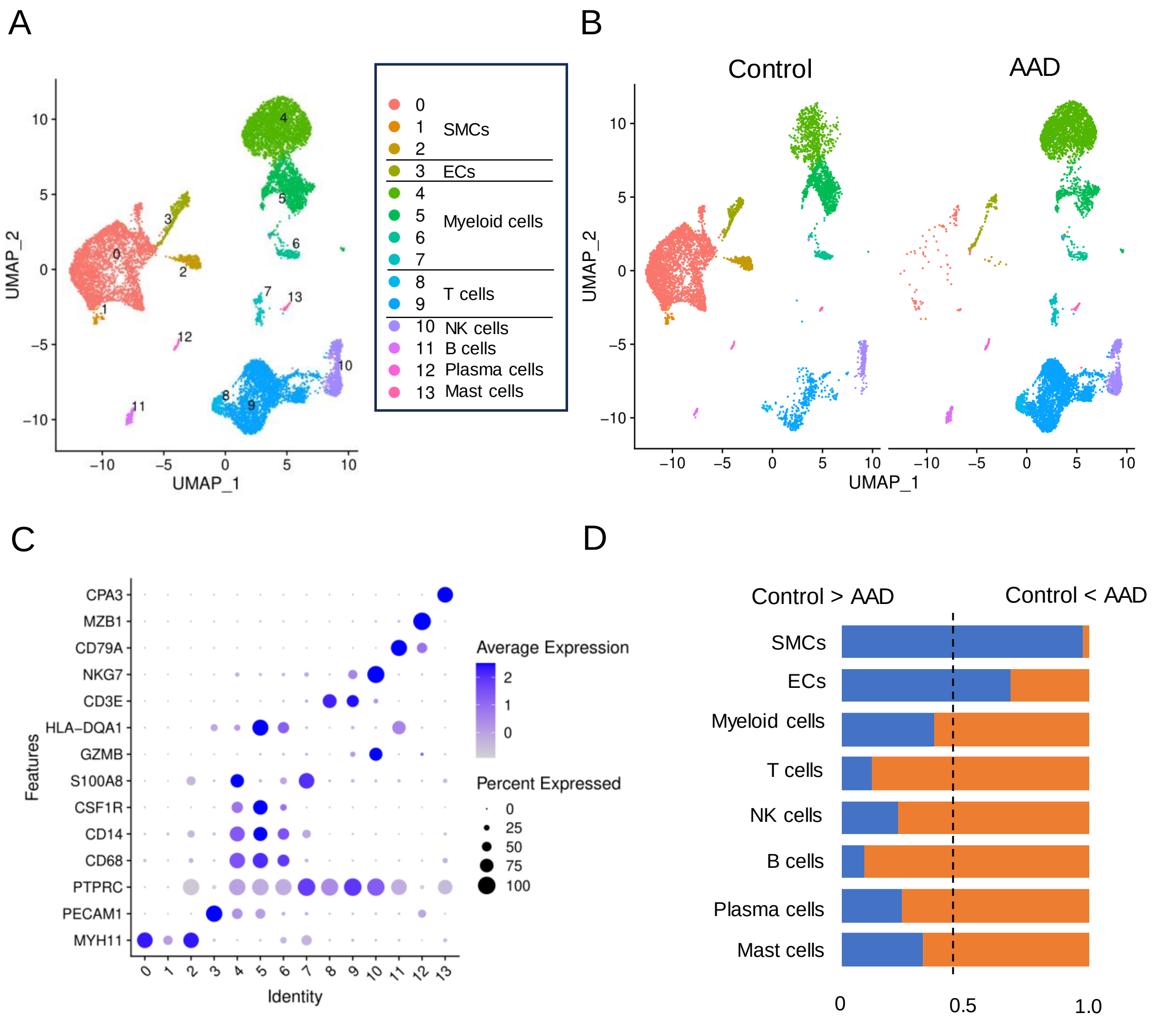

Supplementary Figure 1. Single cell RNA sequencing (scRNAseq) reveals all of the cell populations of ascending aorta in patients with Stanford type A aortic dissection (AAD).
A and B) UMAP dimensionality reduction analysis identifying a unique single cell landscape in aortas of combined populations (A), and each of controls (Control) or AADs (AAD, n = 2) (B). C, Dot plots displaying the signature cell gene expression markers of all of the cell cluster. D, Proportions of each cell cluster between Control and AAD.

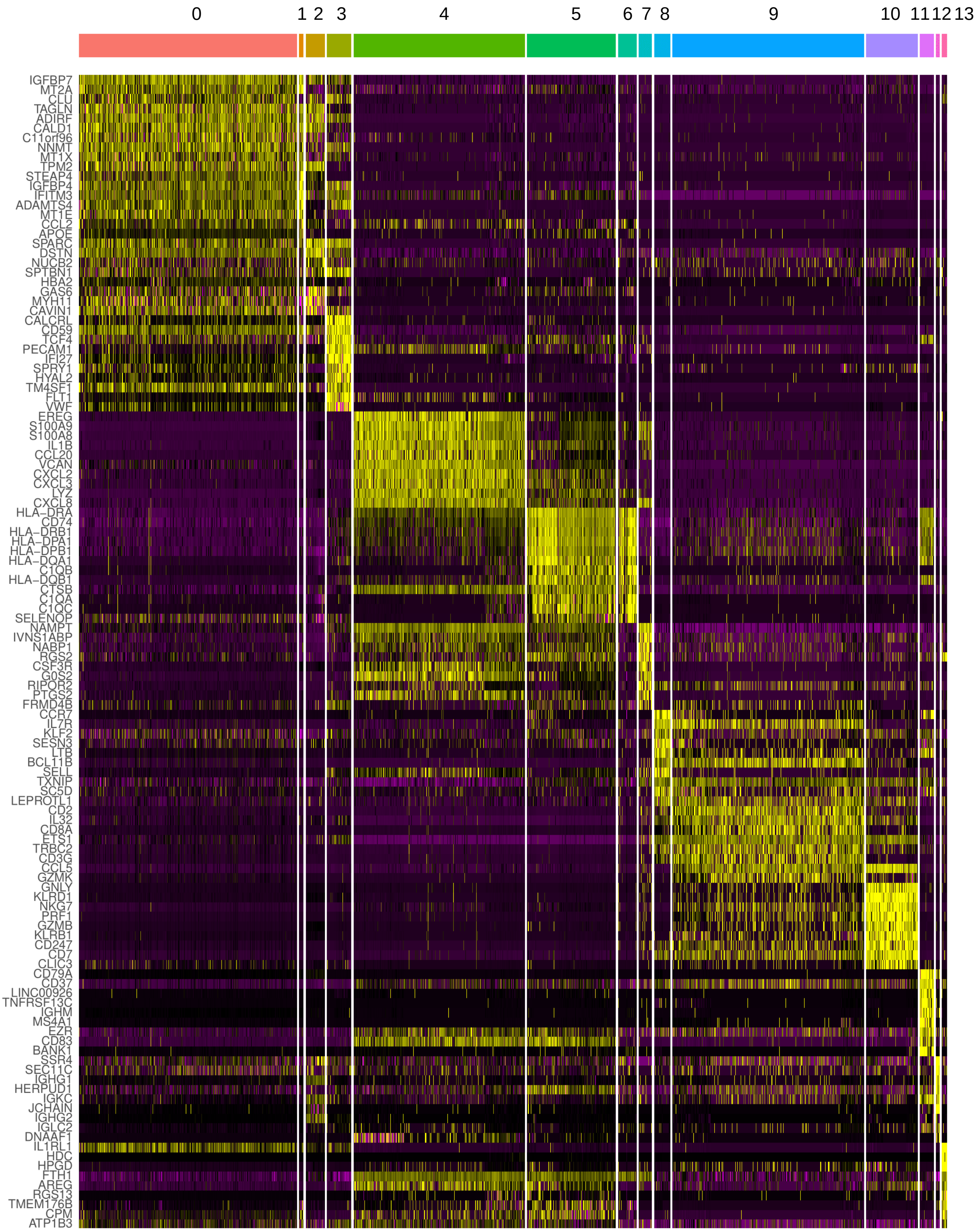

Supplementary Figure 2. Heat map of the top 10 differentially expressed genes in all of the cell populations of ascending aortas in patients with Stanford type A aortic dissection (AAD) (related to Figure. S1).

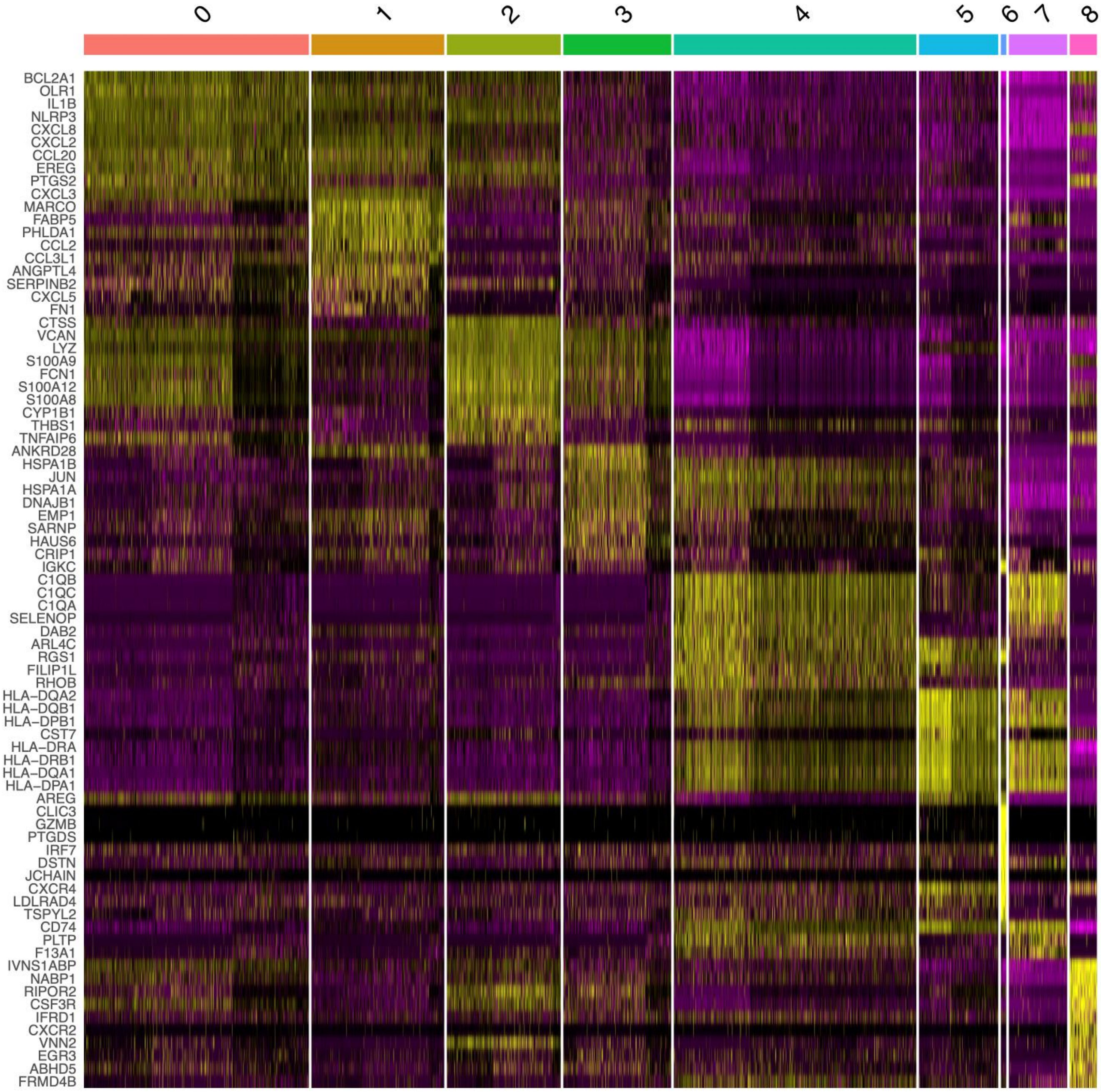

Supplementary Figure 3. Heat map of the top 10 differentially expressed genes in myeloid cell populations. of ascending aortas in patients with Stanford type A aortic dissection (AAD) (related to Figure 2).

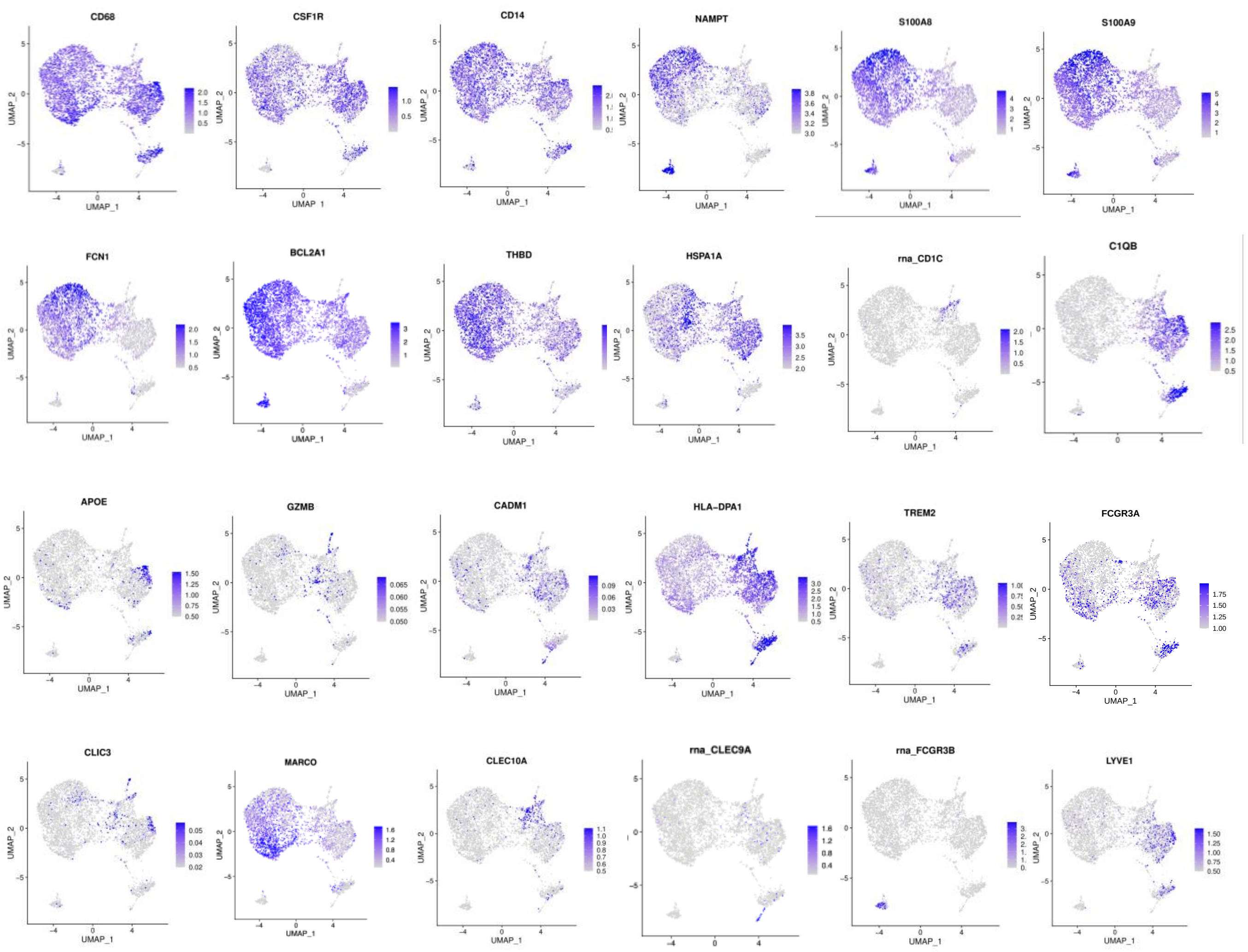

Supplementary Figure 4. Featured plots displaying the additional signature cell gene expression markers in each myeloid cell population of ascending aorta in patients with Stanford type A aortic dissection (AAD) (related to Figure 2C).

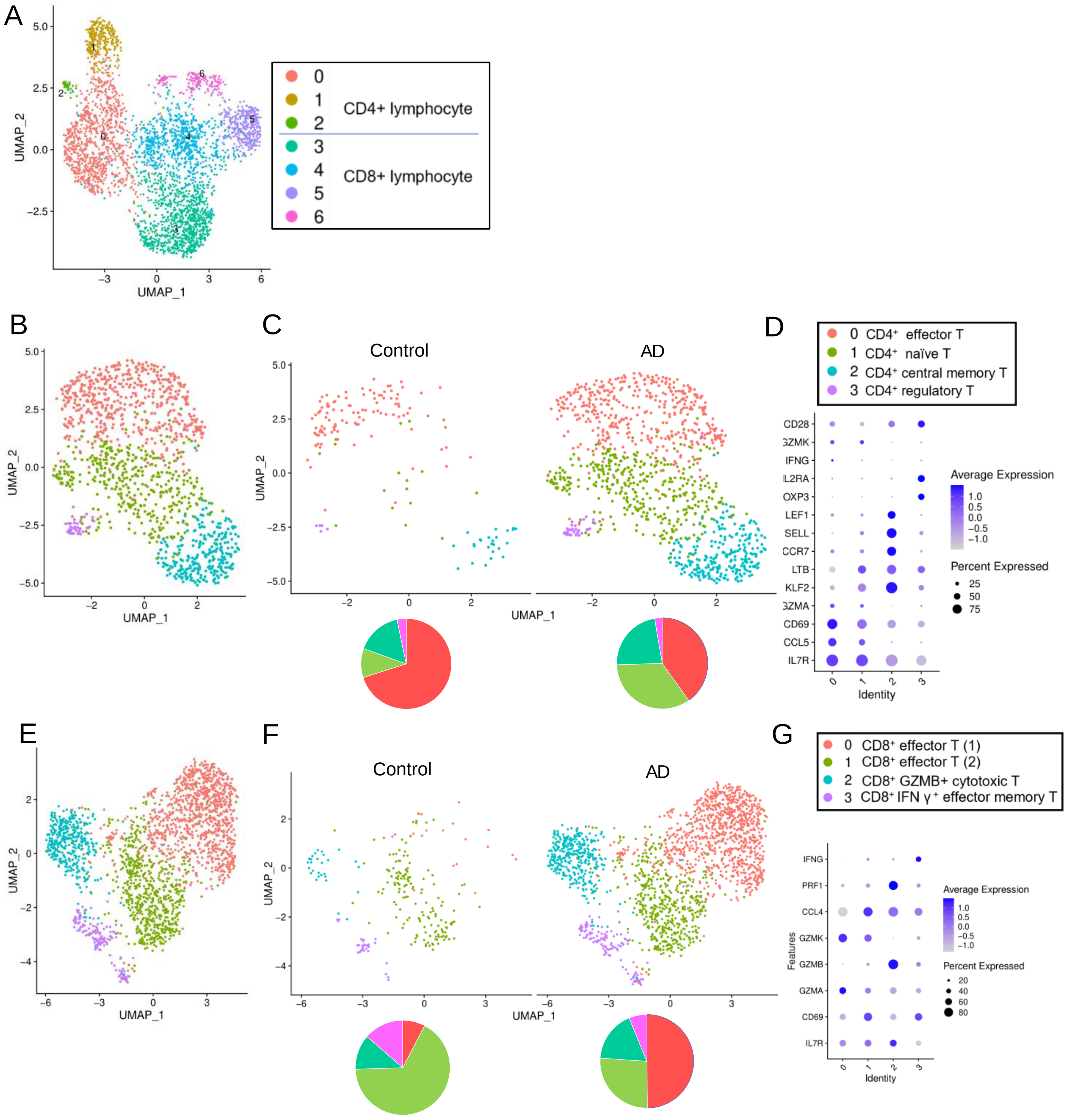

Supplementary Figure 5.

Sub-clustering of T cells shows the emergence of memory T cells in in of ascending aorta in patients with Stanford type A aortic dissection (AAD).

A, All of the T cells which were divided into $CD4^+$ T cells and $CD8^+$ T cells. **B and C**, Sub-clustering of $CD4^+$ T cells by UMAP in combined populations (B) and in each Control or AAD (C, top). Proportions of each $CD4^+$ T cell sub-cluster in each Control or AAD (C, bottom) **D,** Dot plots displaying signature cell gene expression markers for each $CD4^+$ T cell sub-cluster. **E and F**, Sub-clustering of $CD8^+$ T cells by UMAP in combined populations (B) and in each Control or AAD (F, top). Proportions of each $CD8^+$ T cell sub-cluster in each Control or AAD (F, bottom) **G,** Dot plots displaying signature cell gene expression markers for each $CD8^+$ T cell sub-cluster.

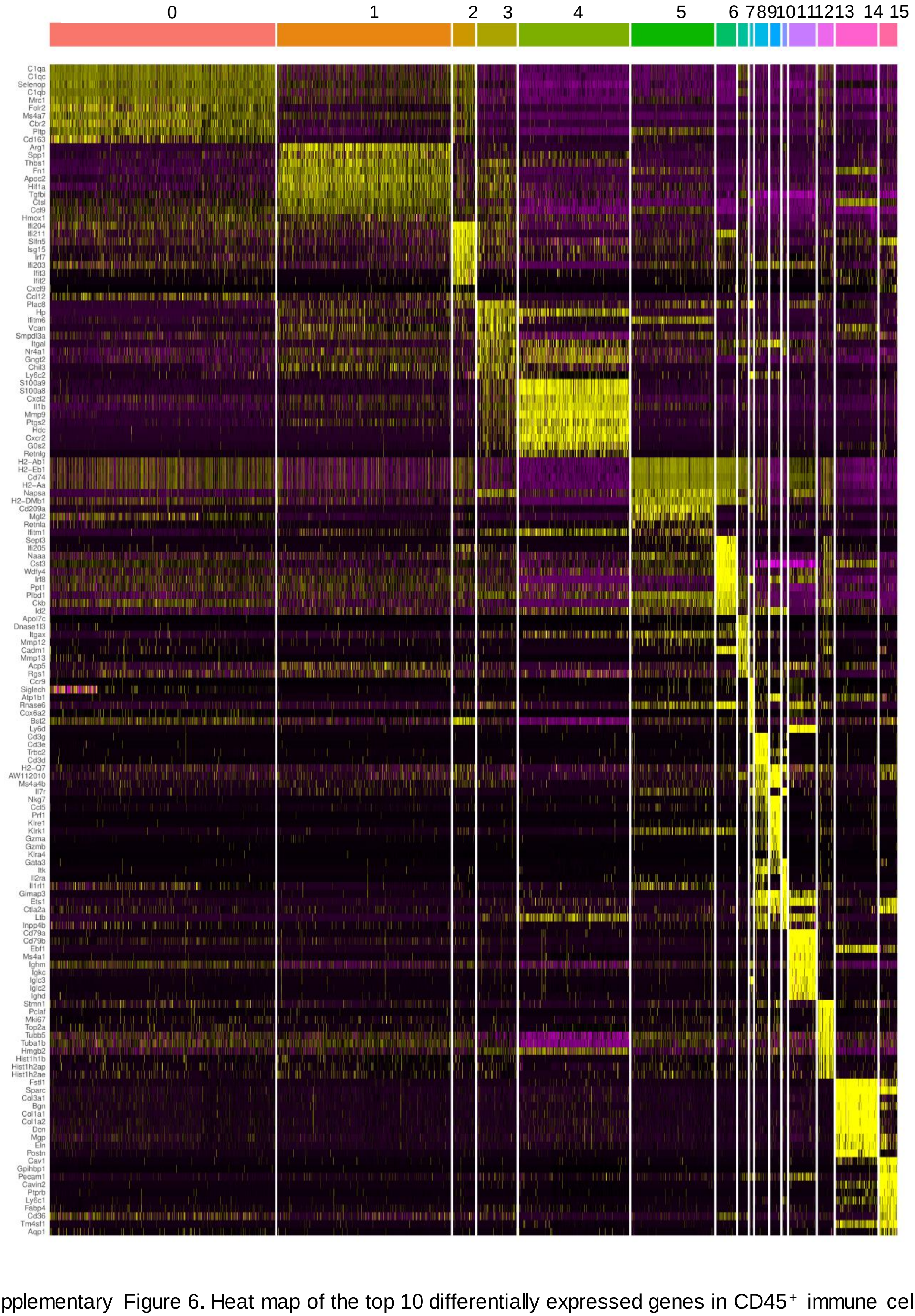

Supplementary Figure 6. Heat map of the top 10 differentially expressed genes in CD45⁺ immune cell populations in a murine AAD model (related to Figure 4).

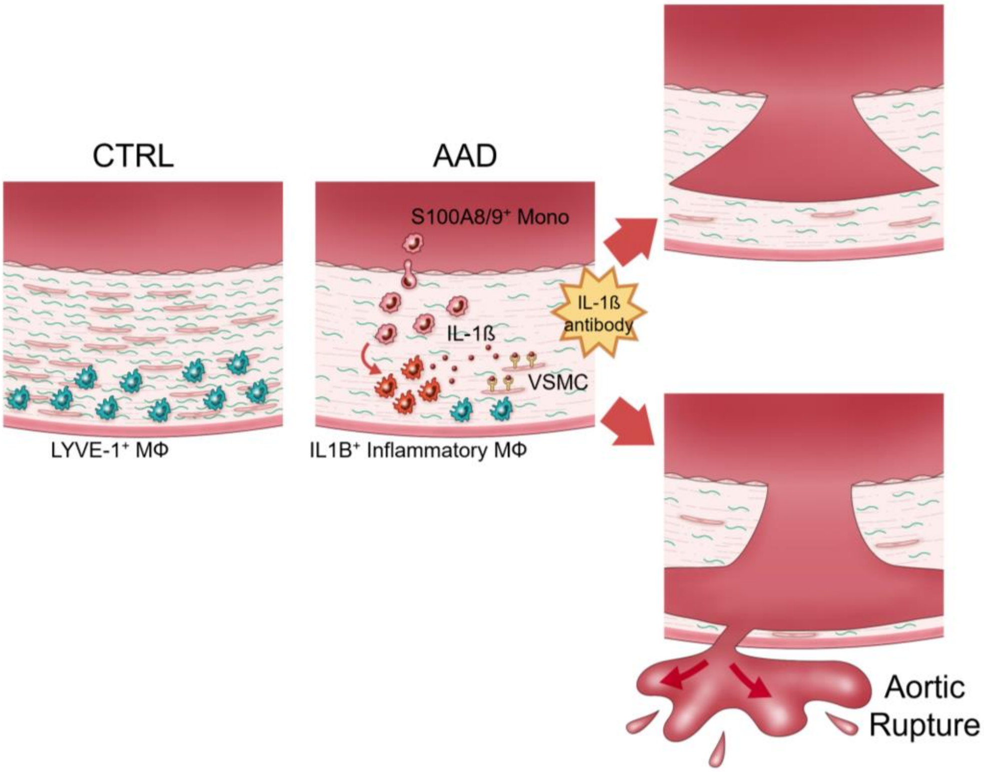

Supplementary Figure 7. Summary of the mechanisms how innate immune systems influence the rupture in acute aortic dissection. S100A8/9^{+} monocytes infiltrated within the aorta and differentiated into IL1B^{+} macrophages. These macrophages affected vascular smooth muscle cells, inhibiting elastin synthesis and causing vascular vulnerability. Inhibition of IL-1β prevented aortic wall degradation and suppressed rupture occurrence in aortic dissection.
